# Priming With the Green Leaf Volatile (Z)-3-Hexeny-1-yl Acetate Enhances Salinity Stress Tolerance in Peanut (*Arachis hypogaea* L.) Seedlings

**DOI:** 10.3389/fpls.2019.00785

**Published:** 2019-06-20

**Authors:** Shufei Tian, Runze Guo, Xiaoxia Zou, Xiaojun Zhang, Xiaona Yu, Yuan Zhan, Dunwei Ci, Minglun Wang, Yuefu Wang, Tong Si

**Affiliations:** ^1^ Shandong Provincial Key Laboratory of Dryland Farming Technology, College of Agronomy, Qingdao Agricultural University, Qingdao, China; ^2^ Shandong Peanut Research Institute, Qingdao, China

**Keywords:** green leaf volatiles, Z-3-HAC, priming, salinity stress tolerance, peanut

## Abstract

Green leaf volatiles play vital roles in plant biotic stress; however, their functions in plant responses to abiotic stress have not been determined. The aim of this study was to investigate the possible role of (Z)-3-hexeny-1-yl acetate (Z-3-HAC), a kind of green leaf volatile, in alleviating the salinity stress of peanut (*Arachis hypogaea L.*) seedlings and the underlying physiological mechanisms governing this effect. One salt-sensitive and one salt-tolerant peanut genotype were primed with 200 μM Z-3-HAC at the 4-week-old stage before they were exposed to salinity stress. Physiological measurements showed that the primed seedlings possessed higher relative water content, net photosynthetic rate, maximal photochemical efficiency of photosystem II, activities of the antioxidant enzymes, and osmolyte accumulation under salinity conditions. Furthermore, the reactive oxygen species, electrolyte leakage, and malondialdehyde content in the third fully expanded leaves were significantly lower than in nonprimed plants. Additionally, we found that application of Z-3-HAC increased the total length, surface area, and volume of the peanut roots under salinity stress. These results indicated that the green leaf volatile Z-3-HAC protects peanut seedlings against damage from salinity stress through priming for modifications of photosynthetic apparatus, antioxidant systems, osmoregulation, and root morphology.

## Introduction

As an important cash and oilseed crop, peanut (*Arachis hypogaea* L.) is widely cultivated in most tropical, subtropical, and temperate regions worldwide (Sharma et al., 2016; Cui et al., 2018). Peanut is also a great source of many nutrients for humans, such as protein, fatty acids, and vitamins (King et al., 2008; Aninbon et al., 2016). Soil salinity is one of the key environmental factors that affects plant growth and reduces crop productivity worldwide (Tanji, 2002; Hasegawa, 2013). More than 800 million hectares of agricultural land have been impaired by salinity (Rengasamy, 2010). Among all types of salinity, the most soluble and widespread salt is sodium chloride (NaCl). Similar to many other leguminous crop species, peanut is moderately sensitive to salinity, especially NaCl stress (Greenway and Munns, 1980). Salinity stress has a severe impact on the growth and morphogenesis of peanut, decreasing seed germination and dry matter accumulation, affecting the establishment of seedling morphology, and inducing damage to the photosynthetic apparatus (Mäser et al., 2002; Deinlein et al., 2014; Yi et al., 2015; Meena et al., 2016).

Plants employ ubiquitous mechanisms to cope with salinity and minimize salt toxicity. The plant responses to salinity stress include the induction of phytohormones and antioxidant systems, vacuole compartmentalization of toxic ions, and synthesis and accumulation of compatible compounds to osmotically balance the cytosol with vacuoles (Cheeseman, 1988; Zhu, 2002; Munns and Tester, 2008; Garma et al., 2015; Ferchichi et al., 2018; Abdel Latef et al., 2019). In the past several decades, plant growth-regulating substances have been widely used to confer salinity stress in many crop species, including sodium selenate (Subramanyam et al., 2019), melatonin (Li et al., 2017; Chen et al., 2018), hydrogen peroxide (Li et al., 2011), brassinosteroid (Divi et al., 2010; Zhu et al., 2015), nitric oxide (Sun et al., 2014; Ahmad et al., 2016), and glycine betaine (Nawaz and Ashraf, 2010; Nusrat et al., 2014; Kreslavski et al., 2017; Annunziata et al., 2019). In addition, traditional breeding and genetic engineering have also been promising approaches for the acquisition of salinity stress tolerance of crops (Hanin et al., 2016; Ismail and Horie, 2017). Although these strategies are well accepted by farmers, more eco-friendly plant growth-regulating substances that confer crop salinity tolerance are required to achieve the goal of agricultural sustainability.

Biogenic volatile organic compounds (VOCs) mainly consist of terpenes, fatty acid-derived products, and products of the shikimic acid pathway, which are emitted by plants under stress (Dudareva et al., 2006; Heil and Silva Bueno, 2007; Nguyen et al., 2016). VOCs can act as an alarm signal when plants are under attack from insect herbivores. Green leaf volatiles (GLVs) are an important group of VOCs for priming plant defenses against insect herbivore attacks, which were first reported by Engelberth et al. (2004). Typically, GLVs are released by plants after mechanical wounding or herbivore attack and could induce defense-related genes to alert the undamaged tissues in plant biotic stress responses (Pare and Tumlinson, 1997; Arimura et al., 2002; Yan and Wang, 2006). However, the role that GLVs play in plant abiotic stress remains an open question. Previous studies, including our research, documented the importance of wounding- or herbivore-induced phytohormones, such as ethylene (ETH) and jasmonic acid (JA), and signaling molecules, such as hydrogen peroxide (H_2_O_2_) and nitric oxide (NO), in response to plant abiotic stress (León et al., 2001; Schilmiller and Howe, 2005; Chauvin et al., 2012; Ahmad et al., 2016; Si et al., 2017, 2018); thus, GLVs might also be a crucial molecule in plant abiotic stress.

GLVs are synthesized *via* the lipoxygenase pathway, where (Z)-3-hexen-1-al (Z-3-HAL), (Z)-3-hexen-1-ol (Z-3-HOL), and (Z)-3-hexeny-1-yl acetate (Z-3-HAC) are all major components. Extensive studies have demonstrated that Z-3-HAC plays a pivotal role in plant defenses against insect herbivore attack (Matsui et al., 2012; Ameye et al., 2015). However, the literature regarding priming by Z-3-HAC in response to plant abiotic stress remains scarce. More recently, Cofer et al. (2018) reported that exogenous Z-3-HAC treatment determines increased growth and reduced damage under cold stress in maize (*Zea mays*) seedlings. This report was the first to describe the priming effects of Z-3-HAC in plant abiotic stress. Given these findings, we speculate that Z-3-HAC could also play a role in other plant abiotic stresses, such as salinity stress. To date, Z-3-HAC has been tested only on maize, but not on other species monocots or dicots. Therefore, a new study was designed in this paper to further our understanding of the role that Z-3-HAC plays in plant abiotic stress. It was hypothesized that exogenous application of Z-3-HAC could enhance salinity stress tolerance in peanut seedlings. This effort to improve salt tolerance in peanut will reduce the yield losses caused by salinity stress, and we can obtain greater output from salinized agricultural land worldwide.

## Materials and Methods

### Plant Materials

Two peanut cultivars, Huayu 20 (abbreviated here as “HY20”) and Huayu 22 (abbreviated here as “HY22”), which are classified as salt-sensitive and salt-tolerant genotypes, respectively, were used as the experimental materials in this study. The seeds were surface sterilized with 2% (v/v) sodium hypochlorite, rinsed two times with tap water, and soaked in tap water overnight. Then, the seeds of uniform sizes were germinated in vermiculite in the dark at 28°C for 2 days before transfer to pots (inner diameter of 9 cm and height of 8 cm with small holes at the bottom, one seedling/pot) filled with 200 g of garden soil each. The seedlings were then transferred to an artificial climate-controlled chamber with an air temperature of 25°C, a light/dark cycle of 16/8 h, a humidity of 60%, and a photosynthetic photon flux density (PPFD) of 1,200 μmol m^−2^ s^−1^. Each pot was watered with 200-ml distilled water on every alternate day. Four-week-old seedlings with uniform sizes were selected for the subsequent experiments.

### Experimental Design

The information of (Z)-3-hexeny-1-yl acetate (Z-3-HAC) (≥98%, Sigma-Aldrich, Inc. USA) were as follows: CAS number of 3,681-71-8, linear formula of CH_3_CO_2_CH_2_CH_2_CH═CHC_2_H_5,_ and molecular weight of 142.20. All selected seedlings were randomly divided into two batches. A half batch of the seedlings was first foliar applied with 200 μM Z-3-HAC (Z-3-HAC was dissolved in 95% (v/v) ethanol as stock solution) twice with a 3-day interval. At the same time, the other half batch was treated with distilled water with the equivalent amount of ethanol. A relatively moderate concentration of Z-3-HAC at 200 μM was most effective according to our previous experiments (data not shown). Seven days after pretreatment, half of the seedlings treated with Z-3-HAC and distilled water were exposed to NaCl stress treatments. Each pot was watered with 200-ml NaCl solution at a concentration of 300 mM three times with a 2-day interval, while the rest of the seedlings were watered with distilled water at the same time. The final salt content of the NaCl-treated soil was 0.35% (w/w), which could be classified as severely saline soil. In total, four treatments were composed: control (water + water without NaCl), Z-3-HAC (Z-3-HAC + water without NaCl), NaCl (water + water with NaCl), and Z-3-HAC + NaCl (Z-3-HAC + water with NaCl). Physiological and biochemical parameters were determined at 7 days after the onset of salinity stress treatment. One representative pot was selected from at least 10 similar-looking plants for each treatment, and pictures were taken. For all the measurements, the third fully expanded leaves from the plant tops were selected. Three independent biological replicates were performed for each treatment.

### Measurement of Shoot Weight and Root Morphology

The seedlings were washed twice with distilled water, the topical moisture was removed, and then the fresh weights of the dissected shoots were measured immediately. To obtain the dry weights, the dissected shoots were oven-dried at 105°C for 15 min to deactivate enzymes and then heated in a stove at 85°C until constant weights were recorded. Meanwhile, the fresh roots were also dissected, carefully washed twice with distilled water, and then scanned using a dual lens scanning system (V700, SEIKO EPSON CORP., Japan) according to the method of Jiang et al. (2017). The data obtained were analyzed using the WinRHIZO Pro software (Version 2012b, Regent Instruments Inc., Canada). There were three independent biological replicates for each treatment and one representative picture is shown.

### Determination of Gas Exchange Parameters, Chlorophyll Fluorescence and Total Chlorophyll Content

Determination of gas exchange parameters was conducted between 9:00 am and 11:00 am using the portable photosynthesis system (Li-COR 6800, Lincoln, NE, USA). The net photosynthetic rate (Pn), stomatal conductance (Gs), intercellular CO_2_ concentration (Ci), and transpiration rate (Tr) were measured based on the following conditions in the leaf chamber: air temperature of 25°C, air relative humidity of 80%, CO_2_ concentration of 400 μmol mol^−1^, and PPFD of 1,000 μmol m^−2^ s^−1^.

Chlorophyll fluorescence was measured after a 30-min dark adaptation period with an imaging pulse amplitude modulated (PAM) fluorimeter (IMAG-MAXI; Heinz Walz, Effeltrich, Germany), as described in detail by Ahammed et al. (2013). The minimum fluorescence emission signal (Fo), maximal fluorescence (Fm), steady-state fluorescence yield (Fs), and light-adapted maximum fluorescence (Fm′) were recorded as the area of interest in the compound leaves. Then, the maximal photochemical efficiency of photosystem II (PSII) (Fv/Fm), the quantum efficiency of PSII photochemistry (ΦPSII), the photochemical activity of PSII (Fv′/Fm′), and the non-photochemical quenching (*NPQ*) were calculated according to the formulas as described by Kramer et al. (2004). The images of Fv/Fm were also exported, and the representative leaf for each treatment is shown.

For the assay of the total chlorophyll content, 0.1 g of fresh leaf was extracted in 25 ml of anhydrous ethanol and acetone (1:1, v/v) solution and incubated for 12 h in the dark at room temperature. Then, the total chlorophyll content (mg g^−1^ FW) was determined colorimetrically at 647 and 663 nm and calculated as originally described by Lichtenthaler and Wellburn (1983).

### Measurement of Relative Water Content, Electrolyte Leakage, and Lipid Peroxidation

The leaf relative water content (RWC) was measured based on the method of Jensen et al. (2000) with some modifications. In total, the leaves were excised and fresh weight (FW) was measured. Then, the leaves were soaked in tubes with 5 ml of deionized water for 4 h at room temperature before the turgid weight (TW) was recorded. Dry weight (DW) was further measured after the leaves were oven-dried for 24 h at 90°C. RWC was calculated by RWC (%) = [(FW − DW)/(TW − DW)] × 100.

The measurement of relative electrolyte conductivity (REC) was conducted using the method of Griffith and McIntyre (1993). The leaf samples were excised immediately and rinsed briefly with deionized water and soaked in 10 ml of deionized water at room temperature for 12 h. The conductivity (C1) was then measured with a conductivity bridge (DDS-307A, LEX Instruments Co., Ltd., China). Then the solution was boiled for 30 min, and the conductivity (C2) was further recorded after cooling. RWC was calculated by REC (%) = C1/C2 × 100.

The lipid peroxidation level was determined by quantifying the equivalents of malondialdehyde (MDA). The 2-thiobarbituric acid (TBA) reaction was used in this assay, and the absorbance values of the red adduct at 450, 532, and 600 nm were recorded to calculate the MDA equivalents as described previously (Hodges et al., 1999). All spectrophotometric assessments in this paper were carried out using a UV–Vis spectrophotometer (UV3200, Mapada Instruments Co., Ltd., China).

### Histochemical Staining and Quantitative Assay of H_2_O_2_ and O_2_^−^

Hydrogen peroxide (H_2_O_2_) in leaves was visually detected by histochemical staining according to the method of Thordal-Christensen et al. (1997) with minor modifications. The leaves were excised from the plants and immediately submerged in 3,3-diaminobenzidine (DAB) solution (1 mg ml^−1^, pH 3.8). Then, the leaves were incubated for 12 h under light with a PPFD of 1,200 μmol m^−2^ s^−1^ at room temperature, after which the leaves were bleached in 95% (v/v) boiling ethanol for approximately 15 min until the brown spots were clearly visualized. Then, the leaves were carefully transferred to fresh 95% (v/v) ethanol, and pictures were taken after cooling. The H_2_O_2_ concentration was determined by measuring the absorbance of the titanium peroxide complex at 410 nm according to the method of Willekens et al. (1997) with minor modifications.

Superoxide anion (O_2_^−^) was also visually detected according to the method originally described by Jabs et al. (1996). In brief, the leaves were excised from the seedlings and soaked in nitro blue tetrazolium (NBT) solution (1 mg ml^−1^, pH 6.1). Then, the leaves were incubated at room temperature in the dark for 6 h before they were completely bleached in 95% (v/v) boiling ethanol. After cooling, the leaves were transferred to fresh ethanol, and pictures were taken immediately. The O_2_^−^ production rate was also quantified according to the previous method of Elstner and Heupel (1976) by monitoring the nitrite formation from hydroxylamine in the presence of O_2_^−^ at an absorbance of 530 nm.

### Extraction and Analysis of Activity of Antioxidant Enzymes

For the determination of antioxidant enzymes, the leaves were frozen immediately in liquid nitrogen and stored at −80°C prior to analysis. In brief, 0.5 g of frozen leaf samples was ground with 5 ml of ice-cold phosphate buffer (50 mM, pH 7.8) containing 20% (v/v) glycerol, 0.2 mM ethylenediaminetetraacetic acid (EDTA), 5 mM MgCl_2_, and 1 mM dithiothreitol (DTT). The homogenates were centrifuged at 4°C for 20 min at 12,000 *g*, and the resulting supernatants were then collected for the determination of enzymatic activity. The total protein content was first analyzed using a Coomassie Brilliant Blue reaction at 595 nm following the method of Bradford (1976). Superoxide dismutase (SOD) activity was assessed by determining its ability to inhibit the photochemical reduction of NBT at 560 nm (Stewart and Bewley, 1980). Guaiacol peroxidase (G-POD) activity was assayed using guaiacol as a substrate at 470 nm as originally described by Cakmak and Marschner (1992). Catalase (CAT) activity was assayed based on the oxidation of H_2_O_2_ and measured as a decline at 240 nm following the method of Patra et al. (1978). Ascorbate peroxidase (APX) activity was determined based on the oxidation of ascorbate and measured as a decline at 290 nm according to the method of Nakano and Asada (1981).

### Contents of Total Soluble Sugars, Sucrose, and Free Amino Acids

Oven-dried (15 min at 105°C and then 85°C for 3 days) leaf samples were powdered with a high-speed ball mill (MM400, Retsch GmbH, Haan, Germany) and mixed thoroughly. A total of 0.1 g of the powder was extracted with 8 ml of 80% (v/v) ethanol in a 10-ml plastic tube at 80°C and centrifuged at 3,000 *g* for 30 min. The supernatant was then collected in a 25-ml glass tube. The extraction was then repeated twice, and the same ethanol was added to the glass tube to a final volume of 25 ml. After mixing thoroughly, the extract was used to determine the contents of total soluble sugars, sucrose, and free amino acids. The anthrone method was adopted, and the absorbance at 620 nm was recorded to calculate the total soluble sugar content according to the method of Buysse and Merckx (1993). For the sucrose content, the resorcinol method was used, which was modified by the method of Buysse and Merckx (1993), and the sucrose content was determined colorimetrically at 480 nm. The content of free amino acids was assessed by the ninhydrin reaction at 570 nm according to the method of Moore and Stein (1954).

### Statistical Analysis

All data collected were statistically analyzed using one-way ANOVA with the SPSS statistical software package (Version 22.0, SPSS Inc., Chicago, IL, USA). Duncan’s test (*p* < 0.05) was performed to evaluate the difference of each treatment. Principal component analysis (PCA) was carried out according to the method of Sun et al. (2018). Each treatment value is the average of three independent biological replicates unless otherwise stated.

## Results

### Effects of Exogenous Z-3-HAC on Plant Growth, Relative Electrolyte Conductivity, and Relative Water Content under Salinity Stress

**Figure 1:**
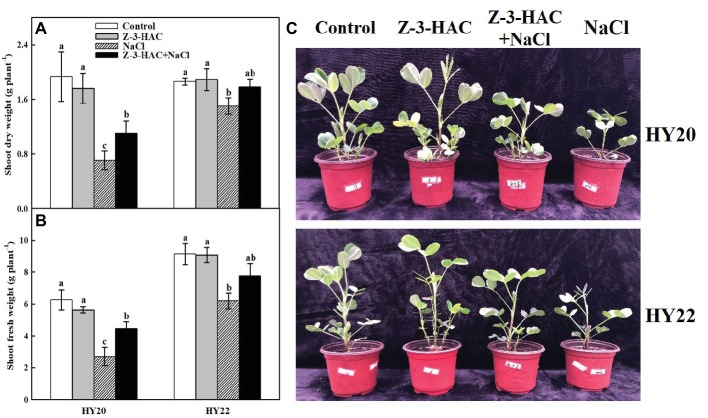
Exogenous Z-3-HAC application conferred salinity stress resistance of peanut seedlings. **(A)** Shoot dry weight, **(B)** shoot fresh weight, and **(C)** growth of peanut seedlings under salinity stress with or without Z-3-HAC priming. The seedlings were primed with distilled water or 200 μM Z-3-HAC twice. After priming, the seedlings were exposed to NaCl stress. At 7 days after the onset of salinity stress treatment, the shoot dry weight and fresh weight were determined, and pictures were taken. Bars are the standard deviations (SD) of three independent replicates (*n* = 3). Error bars labels with different letters indicate significant differences at *p* < 0.05 between treatments according to Duncan’s test.

The first objective was to test the effects of exogenous Z-3-HAC on plant growth. The peanut seedlings were primed with distilled water or 200 μM Z-3-HAC. Then the seedlings were exposed to NaCl stress (NaCl shock did not happen). At 7 days after the onset of salinity stress treatment, the Z-3-HAC-treated seedlings showed a clear apical dominance compared to water-treated seedlings under normal growth conditions in both HY20 and HY22 (Figure 1C). However, no significant difference in the shoot dry weight and fresh weight was observed between these treatments (Figures 1A,B). Exposure of plants to salinity conditions stunted the growth of peanut plants as indicated by the significant decreases in shoot dry weight and fresh weight by 63.39 and 56.94% of HY20 and 19.18 and 32.34% of HY22, respectively. Strikingly, priming with Z-3-HAC resulted in improved plant growth under salinity conditions of HY20, as indicated by the significant increases in shoot dry weight and fresh weight by 55.23and 64.78%, respectively, compared with salinity control. In HY22, Z-3-HAC pretreatment also showed increases in the shoot dry weight and fresh weight by 18.28and 25.48%, respectively, under salinity conditions compared with the salinity control, although the difference was not significant (Figures 1A,B).

**Figure 2:**
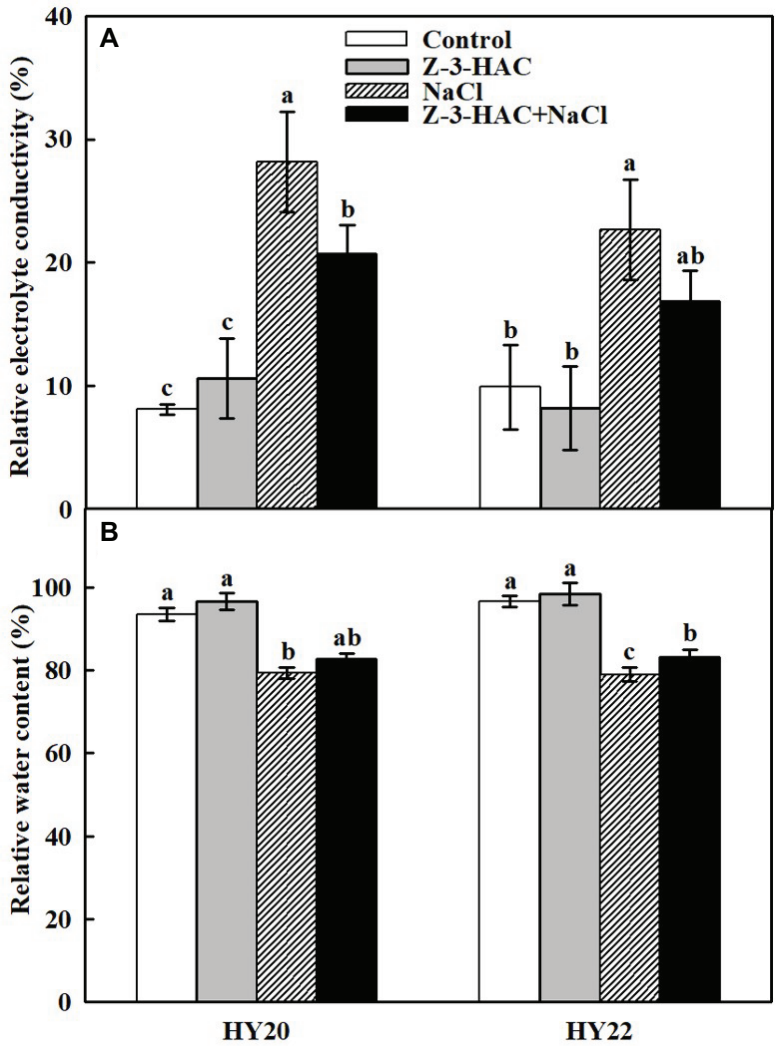
Effects of Z-3-HAC on **(A)** relative electrolyte conductivity (REC) and **(B)** relative water content (RWC) of the third fully expanded leaves in peanut seedlings under salinity stress. The seedlings were primed with distilled water or 200 μM Z-3-HAC twice. After priming, the seedlings were exposed to NaCl stress. At 7 days after the onset of salinity stress treatment, the leaves were excised and the REC and RWC were determined. Bars are the standard deviations (SD) of three independent replicates (*n* = 3). Error bars labels with different letters indicate significant differences at *p* < 0.05 between treatments according to Duncan’s test.

Consistent with the phenotypic changes of the peanut seedlings, exogenous application of Z-3-HAC had no effect on the relative electrolyte conductivity (REC) and relative water content (RWC) of both HY20 and HY22 under normal growth conditions. As expected, salinity stress significantly increased REC by 247.90 and 128.83% in HY20 and HY22, respectively, while decreasing RWC by 15.14and 18.28% in HY20 and HY22, respectively, compared with the control (Figure 2). Notably, priming with Z-3-HAC decreased REC by 36.15 and 34.52% while increasing RWC by 5.5 and 4.3% under salinity stress in severe saline soil compared with their salinity control in HY20 and HY22, respectively.

### Effects of Exogenous Z-3-HAC on Gas Exchange and Chlorophyll Fluorescence Parameters Under Salinity Stress

**Figure 3:**
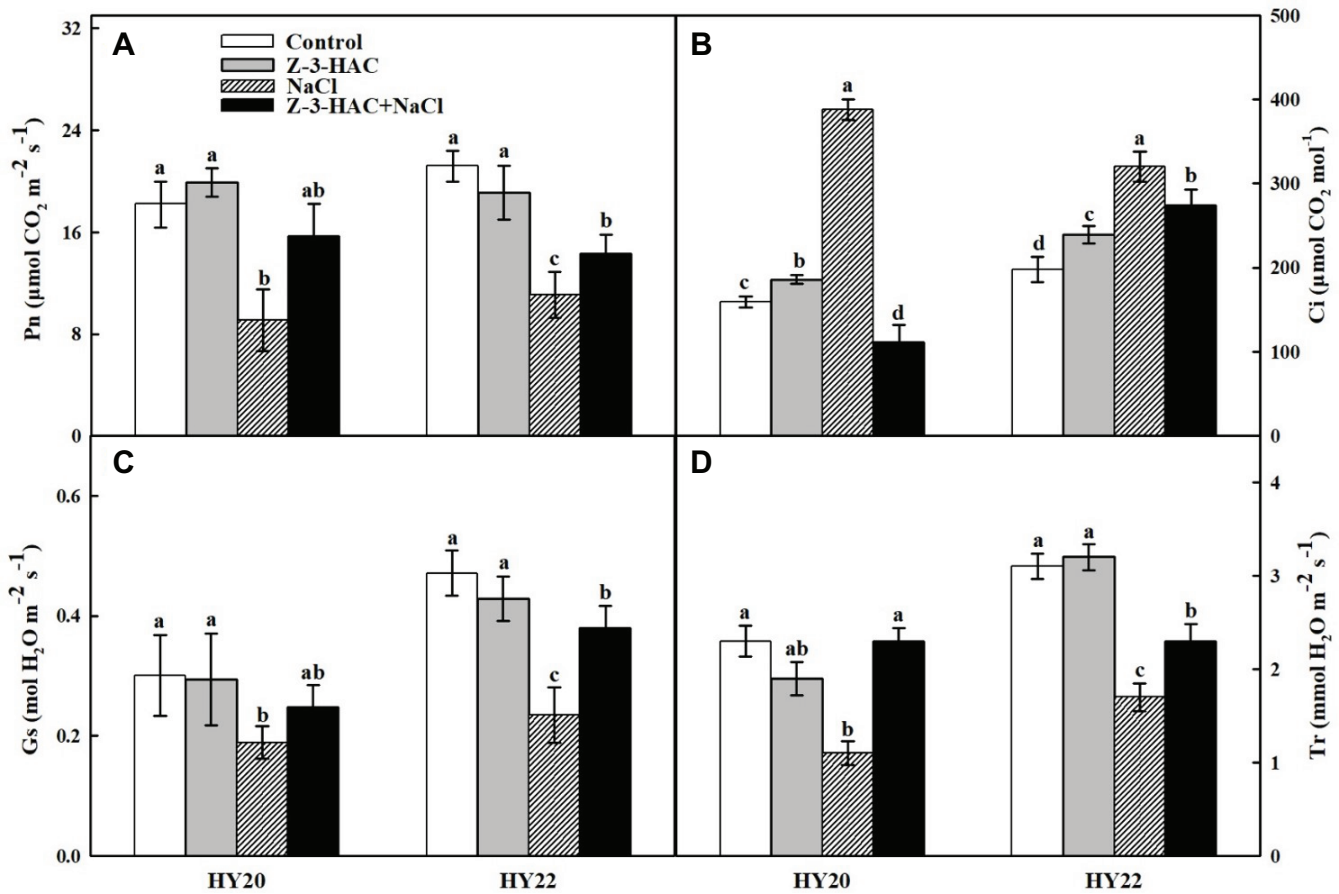
Effects of Z-3-HAC on gas exchange of the third fully expanded leaves in peanut seedlings under salinity stress. **(A)** Net photosynthetic rate (Pn), **(B)** intercellular CO_2_ concentration (Ci), **(C)** stomatal conductance (Gs), and **(D)** transpiration rate (Tr). The seedlings were primed with distilled water or 200 μM Z-3-HAC twice. After priming, the seedlings were exposed to NaCl stress. At 7 days after the onset of salinity stress treatment, the gas exchange parameters were determined. Bars are the standard deviations (SD) of three independent replicates (*n* = 3). Error bars labels with different letters indicate significant differences at *p* < 0.05 between treatments according to Duncan’s test.

Plants treated with only salinity stress displayed significant decreases of 50.00and 47.64% in the net photosynthetic rate (Pn), significant decreases of 37.14and 50.13in the stomatal conductance (Gs), and significant decreases of 52.17and 45.16in the transpiration rate (Tr) in HY20 and HY22, respectively (Figures 3A,C,D), while exhibiting significant increases in the intercellular CO_2_ concentration (Ci) by 144.03and 61.61%, respectively, in HY20 and HY22 compared with the control (Figure 3B). In contrast, exogenous Z-3-HAC significantly reversed the deleterious effects of salinity stress, as indicated by an increase of Pn by 72.52% in HY20 and a significant increase of Pn by 28.83% in HY22, an increase of Gs by 31.03% in HY20 and a significant increase of Gs by 61.77% in HY22, and a significant increase of Tr by 109.09and 35.29%, respectively, in HY20 and HY22, while a significant reduction of Ci by 71.39 and 14.38%, respectively, in HY20 and HY22. The application of exogenous Z-3-HAC alone did not affect Pn, Gs, or Tr in either genotype, whereas Ci was significantly increased by 16.98and 20.71% in HY20 and HY22, respectively.

**Figure 4:**
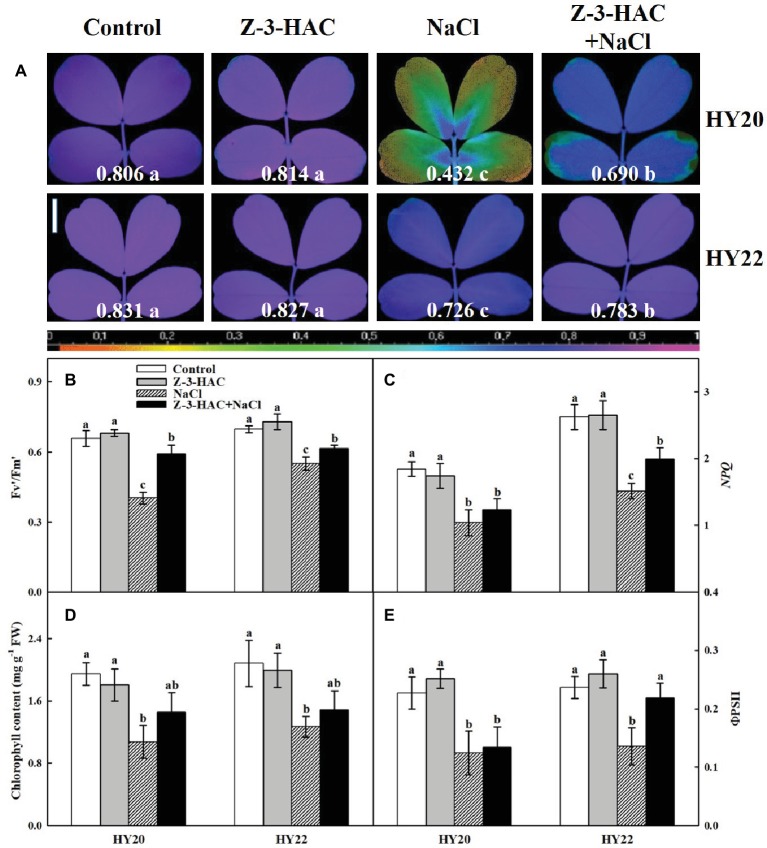
Effects of Z-3-HAC on chlorophyll fluorescence and chlorophyll content of the third fully expanded leaves in peanut seedlings under salinity stress. **(A)** The maximal photochemical efficiency of photosystem II (PSII) (Fv/Fm). The false color code depicted at the bottom of the image ranges from 0 (black) to 1 (purple). The Fv/Fm values are depicted at the bottom of each image. Vertical bar = 1 cm. **(B)** The photochemical activity of PSII (Fv′/Fm′), **(C)** the non-photochemical quenching (*NPQ*), **(D)** the total chlorophyll content expressed in mg g^−1^ FW (fresh weight), and **(E)** the quantum yield of PSII (ΦPSII). The seedlings were primed with distilled water or 200 μM Z-3-HAC twice. After priming, the seedlings were exposed to NaCl stress. At 7 days after the onset of salinity stress treatment, the chlorophyll fluorescence was determined as the area of interest, while the chlorophyll content was also analyzed. Bars are the standard deviations (SD) of three independent replicates (*n* = 3). Images and error bars labels with different letters indicate significant differences at *p* < 0.05 between treatments according to Duncan’s test.

Exogenous Z-3-HAC had no significant effects on the maximal photochemical efficiency of photosystem II (PSII) (Fv/Fm) in both genotypes. Salinity stress significantly decreased Fv/Fm by 86.57and 14.46% in HY20 and HY22, respectively. Again, Fv/Fm was significantly increased by 59.72% in HY20 and 7.85% in HY22 when the seedlings were primed with Z-3-HAC under salinity stress (Figure 4A). Fv/Fm status in different treatments was indicated by pseudo color images of the leaves. Similarly, the other chlorophyll fluorescence parameters, such as the photochemical activity of PSII (Fv′/Fm′), the non-photochemical quenching (*NPQ*), and the quantum efficiency of PSII photochemistry (ΦPSII), displayed similar changes compared with Fv/Fm with a few exceptions where Z-3-HAC failed to increase *NPQ* and ΦPSII under salinity stress in HY20 (Figures 4B,C,E). The leaf chlorophyll content was significantly decreased by 44.84% in HY20 and 39.00% in HY22 under salinity conditions. In contrast, the application of Z-3-HAC showed an insignificant increase in the chlorophyll content by 35.85% in HY20 and 16.78% in HY22 following exposure to salt treatment (Figure 4D).

### Effects of Exogenous Z-3-HAC on ROS Accumulation and Lipid Peroxidation Under Salinity Stress

**Figure 5:**
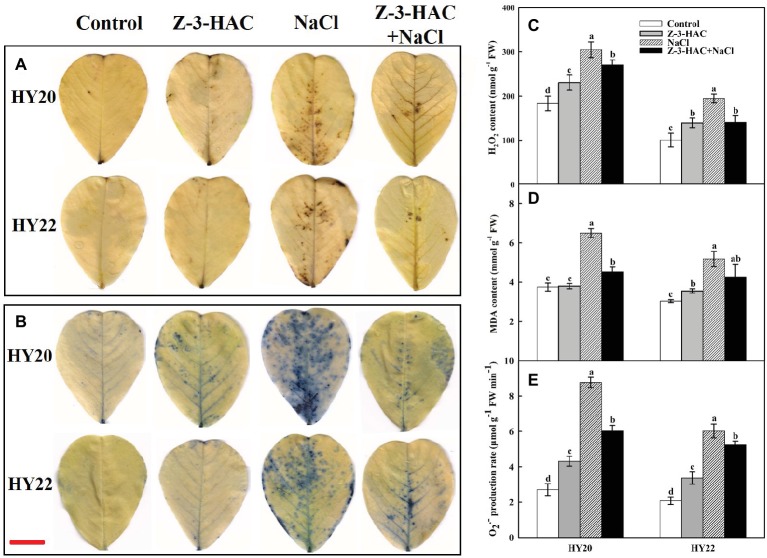
Effects of Z-3-HAC on the accumulation of H_2_O_2_, O_2_^−^, and content of malondialdehyde (MDA) of the third fully expanded leaves in peanut seedlings under salinity stress. The seedlings were primed with distilled water or 200 μM Z-3-HAC twice. After priming, the seedlings were exposed to NaCl stress. At 7 days after the onset of salinity stress treatment, the leaves were excised, and histochemical staining of **(A)** H_2_O_2_ (DAB staining) and **(B)** O_2_^−^ (NBT staining) were performed. Horizontal bar = 1 cm. Meanwhile, the leaves were collected for the determination of **(C)** H_2_O_2_ content, **(D)** MDA content, and **(E)** O_2_^−^ content. Bars are the standard deviations (SD) of three independent replicates (*n* = 3). Error bars labels with different letters indicate significant differences at *p* < 0.05 between treatments according to Duncan’s test.

The accumulations of two representative reactive oxygen species (ROS), H_2_O_2_ and O_2_^−^, were detected using histochemical allocation methods. H_2_O_2_ and O_2_^−^ accumulated slightly following the application of Z-3-HAC under normal conditions. The accumulation of H_2_O_2_ and O_2_^−^ was induced to higher levels under salinity stress but was largely reduced by the exogenous Z-3-HAC in HY20 and HY22 (Figures 5A,B). In keeping with this result, the quantitative data further demonstrated that both H_2_O_2_ and O_2_^−^ were significantly induced by Z-3-HAC and salinity stress in HY20 and HY22. The exogenous application of Z-3-HAC significantly reduced H_2_O_2_ by 11.18% in HY20 and 27.65% in HY22 and significantly reduced O_2_^−^ by 31.20% in HY20 and 13.10% in HY22 under salinity conditions (Figures 5C,E). It is worth noting that the accumulations of H_2_O_2_ and O_2_^−^ were more pronounced in the salt-sensitive genotype HY20 than in the salt-tolerant genotype HY22.

The lipid peroxidation of peanut seedlings was examined according to the accumulation of MDA. Salinity stress significantly induced MDA content by 73.18% in HY20 and 70.32% in HY22. In line with the effect of Z-3-HAC on ROS accumulation, exogenous Z-3-HAC significantly reduced MDA content by 30.39% in HY20 and insignificantly reduced MDA content by 17.51% in HY22 under salinity conditions (Figure 5D). In contrast, priming with Z-3-HAC alone did not affect the MDA content in HY20 but significantly increased the MDA content in HY22 by 16.78% compared with the control.

### Effects of Exogenous Z-3-HAC on Antioxidant Metabolism and Osmolytes Accumulation Under Salinity Stress

**Figure 6:**
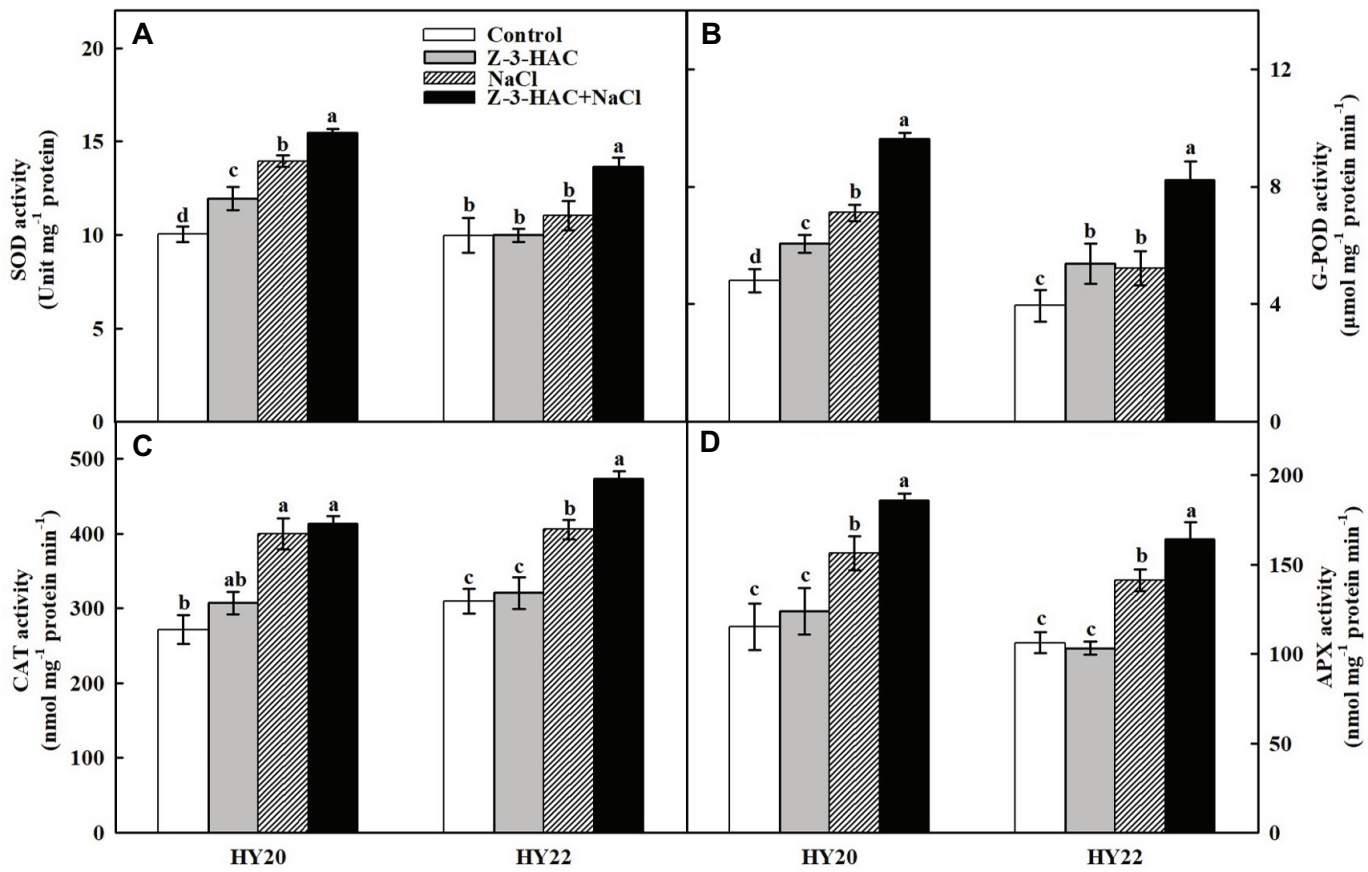
Effects of Z-3-HAC on the activities of the antioxidant enzymes of the third fully expanded leaves in peanut seedlings under salinity stress. The seedlings were primed with distilled water or 200 μM Z-3-HAC twice. After priming, the seedlings were exposed to NaCl stress. At 7 days after the onset of salinity stress treatment, the leaves were collected, and the activities of **(A)** superoxide dismutase (SOD), **(B)** guaiacol peroxidase (G-POD), **(C)** catalase (CAT), and **(D)** ascorbate peroxidase (APX) were analyzed. Bars are the standard deviations (SD) of three independent replicates (*n* = 3). Error bars labels with different letters indicate significant differences at *p* < 0.05 between treatments according to Duncan’s test.

Exogenous application of Z-3-HAC significantly increased the activity of superoxide dismutase (SOD) by 18.86% in HY20, the activity of guaiacol peroxidase (G-POD) by 25.99% in HY20 and 36.45% in HY22 (Figures 6A,B). However, the activities of catalase (CAT) and ascorbate peroxidase (APX) were only slightly affected by sole application of Z-3-HAC in both genotypes (Figures 6C,D). As outlined above, application of Z-3-HAC significantly inhibited the accumulation of MDA during salinity stress. In keeping with these results, exogenous Z-3-HAC resulted in a significant increase in SOD activity by 10.95% in HY20 and 23.65% in HY22, G-POD activity by 35.20% in HY20 and 57.82% in HY22, CAT activity by 26.64% in HY22, and APX activity by 18.99% in HY20 and 16.12% in HY22 under salinity stress compared to the salt treatment control (Figure 6), suggesting that Z-3-HAC treated seedlings had stronger oxidation resistance under salinity conditions.

**Figure 7:**
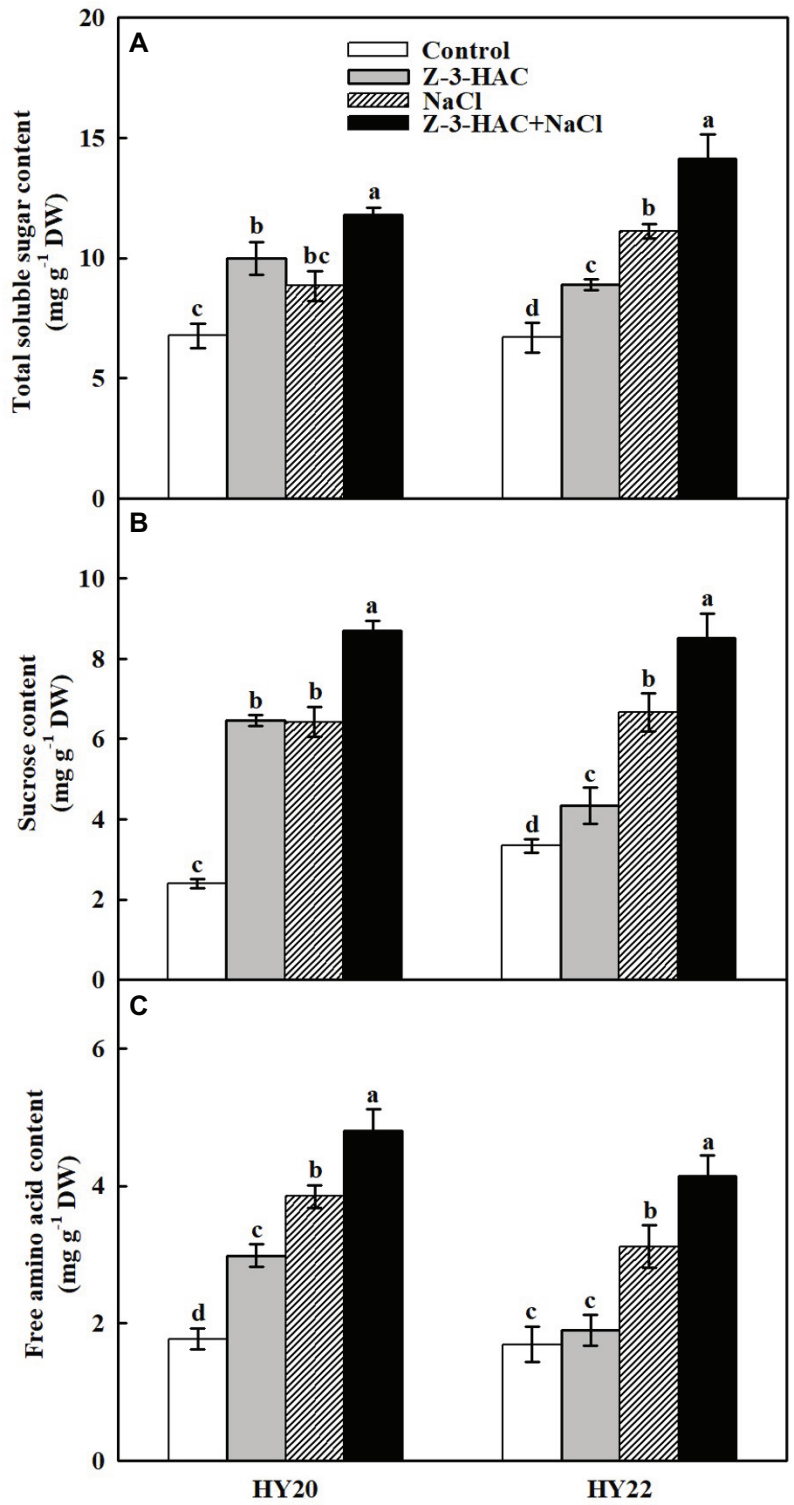
Effects of Z-3-HAC on concentrations of total soluble sugars, sucrose, and free amino acids of the third fully expanded leaves in peanut seedlings under salinity stress. The seedlings were primed with distilled water or 200 μM Z-3-HAC twice. After priming, the seedlings were exposed to NaCl stress. At 7 days after the onset of salinity stress treatment, the leaves were collected, and the concentrations of **(A)** total soluble sugars, **(B)** sucrose, and **(C)** free amino acids were determined. Bars are the standard deviations (SD) of three independent replicates (*n* = 3). Error bars labels with different letters indicate significant differences at *p* < 0.05 between treatments according to Duncan’s test.

Low-molecular weight organic compounds, such as total soluble sugars (TSS), sucrose, and free amino acids (FAA), are major components of plant osmolytes. Both salinity stress and exogenous application of Z-3-HAC significantly increased the concentrations of total soluble sugars, sucrose, and free amino acids in both genotypes. Two exceptions came from the data where salinity stress insignificantly increased the TSS content in HY20, and application of Z-3-HAC failed to increase FAA content in HY22 (Figure 7). In particular, the treatment of “Z-3-HAC + NaCl” had significantly higher concentrations of these osmolytes compared to the salt treatment control, where the total soluble sugar content was increased by 33.41 and 27.17%, sucrose content was increased by 35.36 and 27.63%, and free amino acid content was increased by 24.85 and 32.74% in HY20 and HY22, respectively.

### Effects of Exogenous Z-3-HAC on Root Morphology Under Salinity Stress

**Figure 8:**
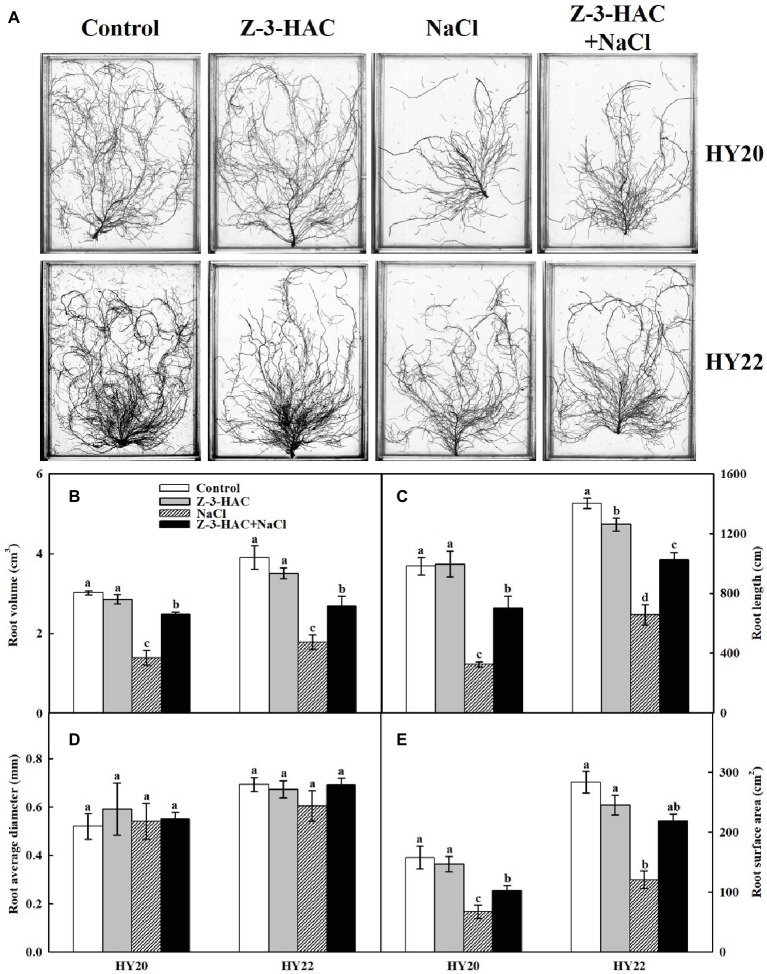
Effects of Z-3-HAC on root morphology of the peanut seedlings under salinity stress. The seedlings were primed with distilled water or 200 μM Z-3-HAC twice. After priming, the seedlings were exposed to NaCl stress. At 7 days after the onset of salinity stress treatment, the roots were washed thoroughly and scanned. **(A)** One representative picture is shown for each treatment. **(B)** Root volume, **(C)** root length, **(D)** root average diameter, and **(E)** root surface area were analyzed using the software. Bars are the standard deviations (SD) of three independent replicates (*n* = 3). Error bars labels with different letters indicate significant differences at *p* < 0.05 between treatments according to Duncan’s test.

To further our understanding of the effects of Z-3-HAC on the underground part of peanut seedlings, the root morphology parameters were determined. Using a dual lens scanning system, we were able to examine the root morphological characteristics of various treatments. From the morphological point of view, salinity stress reduced the total root volume and total root length compared with non-salinity stressed treatments (Figure 8A). The quantitative data further demonstrated that exogenous application of Z-3-HAC did not affect the total root volume, total root length, root average diameter, or the total root surface area in both genotypes compared with the non-salinity stressed control, with only one exception where the total root length was significantly decreased by 10.03% in HY22 (Figure 8C). For the salt-sensitive genotype HY20, the total root volume, total root length, and total root surface area were significantly decreased by 53.9766.90, and 57.44%, respectively, under salinity stress, whereas the magnitude of the reduction was less for the salt-tolerant genotype HY22 than for HY20 (Figures 8B,C,E). The application of Z-3-HAC before salinity stress significantly increased the total root volume by 78.37 and 51.11%, significantly increased the total root length by 116.43 and 56.11%, and increased the total root surface area by 53.23 and 81.39% in HY20 and HY22, respectively, compared to the salt treatment control. However, no significant difference was observed between treatments in root average diameter (Figure 8D).

### Principal Component Analysis

**Figure 9:**
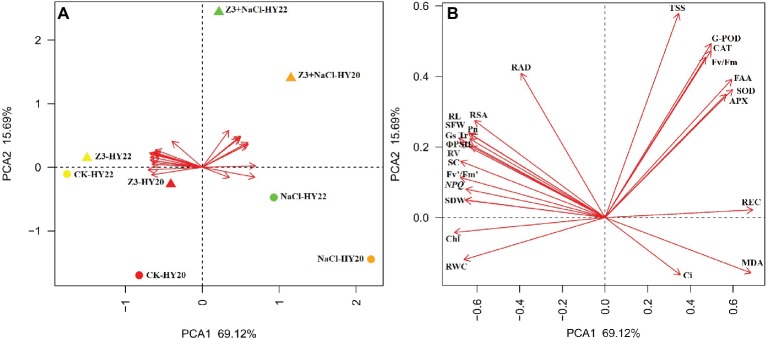
**(A)** Statistical analysis score plot of first and second principal components (PCs) following PC analysis (PCA) based on the parameters collected from four treatments of HY20 and HY22. **(B)** Enlarged view of **(A)** showing the detailed parameters. Arrow direction indicates the correlation among traits, and arrow length indicates the strength of the correlation. Partial abbreviations: SDW, shoot dry weight; SFW, shoot fresh weight; Chl, total chlorophyll content; TSS, total soluble sugars; SC, sucrose; FAA, free amino acids; RV, root volume; RL, root length; RSA, root surface area.

A principal component analysis (PCA) integrating all the information of four treatments (including two cultivars, HY20 and HY22) was performed. The two components of PCA collectively explained 84.81% of data variability. The first PC (PC1) accounted for 69.12% of the total qualitative variation and had REC, SOD, FAA, and APX with high positive loadings. The second PC (PC2) accounted for 15.69% of the total qualitative variation and had TSS, G-POD, CAT, and Fv/Fm with high positive loadings (Figure 9). TSS, G-POD, CAT, Fv/Fm, FAA, SOD, APX, and REC were located toward the positive end of the PC1 axis in the first quadrant. In conclusion, Fv/Fm and the antioxidant system, including the activities of G-POD, SOD, CAT, and APX, were the most important factors in response to Z-3-HAC under salinity stress according to the plot of PC1, PC2, and the treatments in Figure 9A.

## Discussion

It is well accepted that salinity stress markedly inhibits plant growth and adversely affects crop production (Cheeseman, 1988; Munns and Tester, 2008; Deinlein et al., 2014; Niu et al., 2018). In the past decade, plant growth-regulating substances have been widely adapted by research groups to minimize the pernicious effects of salinity stress on crop species, such as silicon (Zhu et al., 2016), melatonin (Arora and Bhatla, 2017; Chen et al., 2018), and epibrassinolide (Wani et al., 2019). Nevertheless, identifying more effective and eco-friendly plant growth-regulating substances is warranted.

A growing body of literature indicates that GLVs are rapidly emitted by plants after wounding to cope with plant biotic stress (Yan and Wang, 2006; Heil, 2014; Tanaka et al., 2018). For a long time, however, scant information was available on the role that GLVs play in the plant abiotic stress response. Recently, Cofer et al. (2018) reported that priming with physiological concentrations of GLV, Z-3-HAC alleviated cold stress in maize seedlings. In the present study, the ameliorative effect of Z-3-HAC in combination with salinity stress in severe saline soil was further investigated using two peanut genotypes. To the best of our knowledge, this is the first time the pivotal role for Z-3-HAC in the plant salinity stress response has been proposed. This mechanism could be of paramount importance to enhance plant salinity stress tolerance and thereby achieve higher crop productivity.

In previous work, Cofer et al. (2018) reported that priming with Z-3-HAC exhibits a positive effect on maize seedlings growth under cold stress. Similarly, the inhibition of growth was clearly relieved by Z-3-HAC application as indicated by plant dry weight and fresh weight when the peanut seedlings were exposed to salinity stress in this study (Figure 1). Notably, the application of Z-3-HAC alone failed to increase or decrease the growth of the peanut seedlings without salinity conditions, suggesting that a moderate concentration of Z-3-HAC could help rescue the seedlings from adverse environmental conditions.

Leaf REC and RWC are vital indicators of plant damage under abiotic stress. The REC increased, while RWC decreased, when plants were suffering from salinity stress (Yi et al., 2015; Zarza et al., 2016; Niu et al., 2018). In keeping with these findings, our results indicated that salinity stress led to an increased level of REC and a decline of RWC in both genotypes. Furthermore, exogenous application of Z-3-HAC could help to maintain the integrity of the plant cell plasma membrane, as evidenced by the decreased REC and increased RWC (Figure 2). In support of the RWC data, the accumulation of osmolytes, such as soluble sugars and free amino acids, was also observed in the “Z-3-HAC + NaCl” treatment (Figure 7). The existence of these substances might also contribute to the higher water content in plant leaves, as previously reported (Koffler et al., 2014; Chen et al., 2018; Wang et al., 2018). The findings to date signified the essentiality of Z-3-HAC in the plant salt response.

Next, we aimed to explore the physiological mechanism of Z-3-HAC in greater detail. The gas exchange parameters indicated that Z-3-HAC effectively attenuated the damage to the photosystem caused by salinity stress in peanut seedlings. An increase in Pn was observed in Z-3-HAC-treated leaves when the seedlings were exposed to salinity stress (Figure 3A). Nevertheless, a considerably steeper reduction of Ci was detected in the “Z-3-HAC + NaCl” treatment compared with salinity stress alone. Thus, the reverse tendency of change in Ci compared to Gs indicated that stomatal limitations were not the rate-limiting factors of Pn when peanut seedlings were exposed to salinity stress (Figures 3B,C). Generally, diffusive (reduction of mesophyll conductance) and metabolic (limitations of photochemistry and related enzymes) processes are involved in nonstomatal limitations (Galmés et al., 2007; Varone et al., 2012). In this paper, the contents of leaf total soluble sugars and sucrose were significantly increased under salinity stress combined with Z-3-HAC treatment (Figures 7A,B), making it likely that the enhanced photosynthesis by Z-3-HAC could be attributed to the acceleration of carbon metabolites (Paul and Pellny, 2003). In addition, soluble sugars and sucrose together with free amino acids are major components of the osmoregulation system, which are interdependently associated with plant salt tolerance (Rai, 2002; Wang et al., 2013; Puniran-Hartley et al., 2014; Gao et al., 2019). The accumulation of these osmolytes was observed in Z-3-HAC-treated seedlings which, in principle, could help to decrease the membrane permeability under salinity conditions (Figure 7). Consequently, the improvement of photosynthetic performance and osmotic accumulation by Z-3-HAC could further increase the plant dry weight, fresh weight, and plant growth (Figure 1), thereby ultimately enhancing salt tolerance in peanut seedlings.

Leaf chlorophyll fluoresce has been principally considered as an important criterion to evaluate the potential injury to photosynthetic apparatus (Xia et al., 2009; Ivanov and Bernards, 2015). The levels of Fv/Fm and Fv′/Fm′ were significantly improved in the “Z-3-HAC + NaCl” treatment, suggesting that Z-3-HAC could reduce the damage to the photosystem under salinity stress in both genotypes (Figures 4A,B). Notably, the induction of Fv/Fm led to an increase in ΦPSII and *NPQ* only in HY22 but not in HY20. The change in ΦPSII could be mainly attributed to the increase in Fv′/Fm′, suggesting that Z-3-HAC could help to accommodate both the lower demand for NADPH and the excessive accumulation of ROS (Figure 4E). The higher level of *NPQ* in the “Z-3-HAC + NaCl” treatment indicated that Z-3-HAC plays an indispensable role in the dissipation of light energy (Figure 4C). The same trend of change in chlorophyll content has been observed in both genotypes, indicating that application of Z-3-HAC helps to minimize the effects of salinity stress on peanut photosynthetic pigments (Figure 4D). These results help to elucidate the profound role of Z-3-HAC in protecting photosynthetic apparatus to combat salinity stress.

The accumulation of ROS has been proven to be a double-edged sword. An accumulating body of evidence documented that the excessive accumulation of ROS could harm the photosystem and plasma membrane, whereas moderate induction of ROS by biotic stress or abiotic stress might be a crucial signal to alert the plants for further response (Neill et al., 2002; Mittler et al., 2004; Miller et al., 2008; Baxter et al., 2014; Qi et al., 2017; Waszczak et al., 2018). We therefore determined the accumulations of two representative ROS, H_2_O_2_ and O_2_^−^ using both histochemical allocation and chemical quantitative analysis methods. The accumulations of H_2_O_2_ and O_2_^−^ were detected in both genotypes under salinity conditions, whereas application of Z-3-HAC largely reduced the ROS level. In addition, the reduction of ROS level was accompanied by the lowered MDA content, indicating that Z-3-HAC enhanced the ROS scavenging capacity in peanut leaves (Figure 5). Interestingly, H_2_O_2_ and O_2_^−^ were also observed after the application of Z-3-HAC under normal growth conditions. The MDA content was barely affected in HY20 but was slightly increased in HY22; however, the increase was not sufficient to cause any damage to the seedlings according to the data in this paper. Thus, we deduce that the ROS induced by Z-3-HAC is more likely to be a signal, rather than a harmful substance, in response to salinity stress. In fact, H_2_O_2_ induced by plant growth-regulating substances has been frequently reported to be involved in plant abiotic signaling responses (Zhou et al., 2014; Xia et al., 2015; Dietz et al., 2016; Choudhury et al., 2017). Therefore, further research is required to elucidate the detailed mechanisms of Z-3-HAC signal transduction.

It is well accepted that SOD catalyzes the disproportionation of singlet oxygen and produces H_2_O_2_ (Li et al., 2015). We observed that salt-sensitive peanut genotype HY20 had higher levels of SOD activity than salt-tolerant peanut genotype after application of Z-3-HAC under normal growth conditions (Figure 6A). In this respect, the greater accumulation of H_2_O_2_ in HY20 might be the result of activated SOD. The alleviating effect of exogenous Z-3-HAC on leaf oxidative stress was further confirmed by the enhanced activities of G-POD, CAT, and APX, where “Z-3-HAC + NaCl” treatment processed higher activities of these antioxidant enzymes compared with other treatments in both genotypes (Figures 6B–D). These results are consistent with the ROS data and support the idea that Z-3-HAC could alleviate leaf oxidative stress by modifying the antioxidant system.

To explore the mechanisms underlying the ameliorating effect of Z-3-HAC on salinity stress-induced root growth inhibition, the root morphology was further characterized. As expected, salinity stress suppressed root growth and reduced the total root volume, total root length, and root surface area. However, the root average diameter was barely affected by salinity stress (Figure 8). Exogenous application of Z-3-HAC significantly induced the total root volume, total root length, and root surface area in both genotypes compared with salinity stress alone treatment, providing unequivocal evidence that the green leaf volatile Z-3-HAC could protect both the aboveground and the underground portion of the seedlings against damage from salinity stress.

In conclusion, our results showed that priming with the green leaf volatile Z-3-HAC attenuated salinity stress-induced photoinhibition and growth inhibition in both salt-sensitive and salt-tolerant peanut seedlings. Exogenous application of Z-3-HAC alleviated the oxidative stress under salinity conditions by enhancing the antioxidant systems, resulting in lower ROS levels compared to the nonprimed seedlings. Additionally, modulation of osmolytes, such as total soluble sugars, sucrose, and free amino acid contents, and modification of root morphology were found to be closely related to the above physiological responses. This study promotes a more comprehensive understanding of the ameliorating functions of green leaf volatiles under salinity stress. Future studies using molecular and proteomic approaches are still required to fully elucidate the role of Z-3-HAC in the plant salinity stress response, as well as the signaling events involved.

## Data Availability

All datasets generated for this study are included in the manuscript.

## Author Contributions

TS, ST, and RG conceived and designed the experiments. XXZ, XJZ, XY, and YZ performed the experiments and analyzed the data. DC, MW, and YW performed the analyses. TS, ST, and RG contributed to the writing of the manuscript, and performed the final editing of the manuscript.

### Conflict of Interest Statement

The authors declare that the research was conducted in the absence of any commercial or financial relationships that could be construed as a potential conflict of interest.
